# Genotator: A disease-agnostic tool for genetic annotation of disease

**DOI:** 10.1186/1755-8794-3-50

**Published:** 2010-10-29

**Authors:** Dennis P Wall, Rimma Pivovarov, Mark Tong, Jae-Yoon Jung, Vincent A Fusaro, Todd F DeLuca, Peter J Tonellato

**Affiliations:** 1 Center for Biomedical informatics, Harvard Medical School, Boston, MA 02115; 2 Department of Biomedical Informatics, Columbia University, New York, NY 10032

## Abstract

**Background:**

Disease-specific genetic information has been increasing at rapid rates as a consequence of recent improvements and massive cost reductions in sequencing technologies. Numerous systems designed to capture and organize this mounting sea of genetic data have emerged, but these resources differ dramatically in their disease coverage and genetic depth. With few exceptions, researchers must manually search a variety of sites to assemble a complete set of genetic evidence for a particular disease of interest, a process that is both time-consuming and error-prone.

**Methods:**

We designed a real-time aggregation tool that provides both comprehensive coverage and reliable gene-to-disease rankings for any disease. Our tool, called Genotator, automatically integrates data from 11 externally accessible clinical genetics resources and uses these data in a straightforward formula to rank genes in order of disease relevance. We tested the accuracy of coverage of Genotator in three separate diseases for which there exist specialty curated databases, Autism Spectrum Disorder, Parkinson's Disease, and Alzheimer Disease. Genotator is freely available at http://genotator.hms.harvard.edu.

**Results:**

Genotator demonstrated that most of the 11 selected databases contain unique information about the genetic composition of disease, with 2514 genes found in only one of the 11 databases. These findings confirm that the integration of these databases provides a more complete picture than would be possible from any one database alone. Genotator successfully identified at least 75% of the top ranked genes for all three of our use cases, including a 90% concordance with the top 40 ranked candidates for Alzheimer Disease.

**Conclusions:**

As a meta-query engine, Genotator provides high coverage of both historical genetic research as well as recent advances in the genetic understanding of specific diseases. As such, Genotator provides a real-time aggregation of ranked data that remains current with the pace of research in the disease fields. Genotator's algorithm appropriately transforms query terms to match the input requirements of each targeted databases and accurately resolves named synonyms to ensure full coverage of the genetic results with official nomenclature. Genotator generates an excel-style output that is consistent across disease queries and readily importable to other applications.

## Background

The promise of personalized and genetic-based medicine has encouraged researchers to develop new technologies to search for genetic causes of common disease. More and more genome data are becoming available due to these technological advances in genotyping and increasing numbers of genome-wide association studies (GWAS). Concomitant with this rise in disease genomics is the rise of publicly accessible databases to report clinical relevance of mutations and provide rankings of genes in terms of their roles in disease. Top among these include several large and established resources like Online Mendelian Inheritance in Man (OMIM) [1], The Human Gene Mutation Database (HGMD) [2], and GeneCards [3] that attempt to cover a wide range of human disease using semi-automated approaches, as well as smaller resources devoted to manual reports of genetic association to high profile diseases, including SFARI Gene for Autism [4], PDGene for Parkinson's Disease (PD) [5], and AlzGene for Alzheimer Disease [6]. The former tend to cover large numbers of human diseases, but also tend to differ, sometimes dramatically, in their concepts of disease-gene association. The latter tend to be rich in content and reliability, but also have a tendency to be incomplete in coverage and lag behind the emerging research trends, at least in part because much of their content is added manually. In addition, the number of such manually curated databases remains restricted to priority diseases for which federal or other funding is available. Many databases are also rarely updated, difficult to navigate, provide information without rankings, maintain inconsistent coverage of information and/or lack a description of their methodology. While all of these resources have merit, a researcher interested in the complete set of the genetic knowledge for a disease or set of diseases is left having to search through a variety of databases to manually compile the results. A resource that automates this process by computationally integrating results from a host of resources to provide best-of-breed genetic knowledge would be a tremendous boon to the field.

Motivated by the need for an integrated system for gene-disease knowledge, we built an engine called Genotator that integrates a wide array of trusted databases and provides a rich set of annotations relating genes to disease. We designed Genotator as a fully automated real-time aggregator of all relevant and up-to-date information about each gene linked to a particular disease. Every disease term queried in the Genotator returns with a list of genes and relevant attributes and annotations for each gene. In the present manuscript we describe the Genotator algorithm and demonstrate its functionality using three use cases, Autism Spectrum Disorder (ASD), Parkinson's Disease (PD), and Alzheimer Disease (AD).

## Implementation

### Databases

We manually examined 33 external databases and identified 11 that had the depth and breadth appropriate for general disease annotation (Table 1). We chose the original 33 based on disease coverage and comprehensiveness and their amenability to automation via screen scraping or other methods. We also chose only those resources that could be queried by disease to return a gene list and associated attributes, such as supporting citations.

**Table 1:** Databases integrated within Genotator (Statistics Gathered: June 19, 2010)

Database	Website	Description	Statistics
GeneCards [3]	[17]	Searchable database of human genes that provides concise genomic, genetic and functional information	73,017 Entries, 28,656 of them with symbols approved by the HUGO gene nomenclature committee
Genetic Association Database [8]	[18]	Archive of human genetic association studies of complex diseases and disorders	2673 genes for 5636 diseases/phenotypes
HuGE Navigator [7]	[19]	Knowledge base including information on gene-disease and gene-gene associations	9429 genes for 2215 diseases
Human Gene Mutation Database [2]	[20]	Database established for the study of mutational mechanisms in human genes	72414 mutations in public release
Online Mendelian Inheritance in Man [1]	[21]	NCBI's compendium of human genes and genetic phenotypes	13, 158 genes for 2799 phenotypes/diseases
Your Favorite Gene	[22]	Database containing scientific descriptions and overviews of genes and their corresponding proteins with links to the most used gene data-banks	~ 8595 genes
UniProt [23]	[24]	Central database of protein sequences with accurate, consistent, rich sequence and functional annotation	UniProtKB/Swiss-Prot Release 2010_06 of 18-May-10 of contains 517100 sequence entries
			UniProtKB/TrEMBL Release 2010_07 of 15-Jun-2010 of contains 11109684 sequence entries
PharmGKB [25]	[26]	Database of primary genotype and phenotype data, annotate gene variants and gene-drug-disease relationships	3,197 diseases, 26,216 genes
Entrez Gene	[27]	Searchable database of genes, from RefSeq genomes, and defined by sequence and/or located in the NCBI Map Viewer	42644 genes
WikiGenes [28]	[29]	Non-profit, open access database of articles on genes, proteins and chemical compounds	Global community
GenAtlas [30]	[31]	Database containing relevant information with respect to gene mapping and genetic diseases	22466 genes/4434 phenotypes

### Algorithm

**Figure 1:**
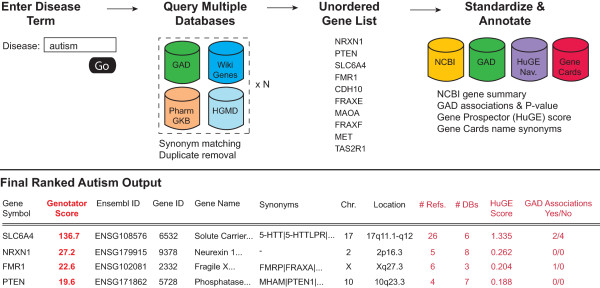
**Genotator algorithm**. A user inputs a disease term into the search field of the Genotator web resource http://genotator.hms.harvard.edu to initiate the pipeline. Genotator then simultaneously queries the databases listed in Table 1 to generate an unordered set of genes while resolving synonyms and removing duplicate entries. The algorithm then gathers characteristics from specific databases, principally including GAD, Gene Prospector (from HuGE Navigator), and NCBI, in order to build an attribute vector and to calculate a relevance score (scoring algorithm detailed in the Implementation section, dark red fields are used in calculating the final Genotator score). The complete set of results, including the Genotator score, associated gene attributes and supporting publications, are displayed directly in the website and made available for download.

We designed an algorithm to query a given disease term in the 11 databases described in Table 1. The code was written in Java and Python and designed to form the union of the lists of genes returned from a disease query while recording whether a gene was present in or absent from each database. We also designed the code to run on a research cluster so that multiple requests could be run in batch on 6 available nodes. The length a single job was dependent on the size of the result set and on the response times of the 11 external resources. As a secondary step, the algorithm queried NCBI with the list of gene symbols to provide an enriched set of annotations including official gene symbol, Ensembl ID, gene ID, gene name, chromosome, location, symbol synonyms, and aliases (Table 2). If separate entries in the results were found to be synonyms, the algorithm collapsed the rows into a single entry under the official NCBI name taking the consensus of the results from all databases queried. The final results were formatted in a single tab-delimited text file for display/download on the Genotator website (example of pipeline provided in Figure 1).

**Table 2:** The characteristics included in Genotator from select resources.

Database	Characteristics Incorporated
Genetic Association Database	List of Genes
	"Yes" Association (published statement of link between gene and disease phenotype)
	"No" Association (published statement of no link between gene and disease)
	p-value (from genome wide association studies)
HuGE Navigator	List of Genes (official gene symbols)
	Gene Prospector Score [9]
	List of PubMed References
Entrez Gene	List of Genes (official gene symbols)
	Official Full Name
	Symbol Synonyms
	Chromosome Number
	Location on Chromosome
	Gene ID
	Ensembl ID

All other databases listed in Table 1 provided a list of genes only.

In addition, we included a select group of high-value parameters to enrich the quality of the data with regards to clinical and experimental relevance of each gene. Specifically, we included from HuGE Navigator [7] and the Genetic Association Database [8] the manually assigned labels "Yes Association", "No Association", "Unknown Association" (where yes association indicates positive support for a gene's role in the disease phenotype) for each gene-article pair, p-values from genome-wide association studies, and the Gene Prospector score of association [9]. All key parameters were then compiled into an attribute vector for scoring and ranking of genes (described in the section immediately below). Finally, to obtain a list of publications supporting a gene-disease pair, we queried HuGE Navigator first with the disease of interest and then separately with the gene name (in this case the official gene symbol) and reported the intersecting publications in the final results.

### Score

The final step of the Genotator algorithm was the implementation of a scoring system to assign the strength of association per gene to the disease of interest. The Genotator score (GS) compiled information from the 11 databases using the following formula.

$$\begin{array}{l} GS=\\ GAD_{Y}-GAD_{N}+\phi (GPS)\\ \;\;+1/\gamma (DB)+1/\kappa (REF) \end{array}$$

*GAD_Y _*= Total number of "Yes" labeled associations for the gene and disease in the Genetic Association Database

*GAD_N _*= Total number of "No" labeled associations for the gene and disease in the Genetic Association Database

*GPS *= Gene Prospector's score of gene-disease association

*DB *= Number of total databases (out of 11) that the gene appeared in

*REF *= Number of total references for the given gene

The constants *ϕ *, *γ *, and *κ *were used to weight the contributions of the GPS, DB, and REF parameters. We elected to set these values at 100, 10, and 5, respectively, as these numbers provided the best overall performance. However, we designed the Genotator software to enable users the ability to alter these parameters should alternative weighting schemes be desired. In addition, Genotator reports the complete set of attributes together with the GS score, thus enabling users the flexibility to retain the original scoring scheme or to define an alternative strategy. We devised the GS to serve as guide for gene prioritization that best captures the history of biomedical research findings while also ensuring adjustability of the threshold for inclusion/exclusion of genes.

### Evaluation

We elected to use three trusted, manually updated, and disease-specific databases to provide (1) a list of vetted candidate genes that have clear association to the disease based on published research (linkage studies, genome-wide association studies, etc.), and (2) an independent ranking of the strength of association between the genes and the disease of interest against which to compare ranked results from Genotator. The three databases, SFARI Gene, PDGene, and AlzGene target Autism Spectrum Disorder (ASD), Parkinson Disease (PD), and Alzheimer's Disease (AD), respectively. SFARI gene is a leading repository of genetic information for ASD that is updated with high frequency by manual curators. PDGene is one of the most trusted web resources for genes associated with PD and has previously been used to validate the Gene Prospector scoring algorithm in Yu et al. [9]. AlzGene [6] has a similar structure to PDGene, and is also widely regarded as an authoritative source of information on the genes associated with AD. To generate a metric for ranking the genes reported by SFARI, we subtracted the total number of negative from the total number of positive associations that were directly reported in the database. PDGene and AlzGene provided a ranking of the top gene candidates in section of their websites called "TopGenes". At the time of writing, PDGene listed 32 and AlzGene listed 40 top genes. In addition, both resources assigned a label of "strong", "moderate", or "weak" to each top gene candidate based upon amount of evidence, extent of replication and protection from bias [10]. By utilizing these resources as a baseline for comparison, we could determine how well the automated procedures employed by Genotator compared to standards of manual annotation, both in terms of coverage of published research as well as in terms of prioritization of gene candidates.

### Website

**Figure 2:**
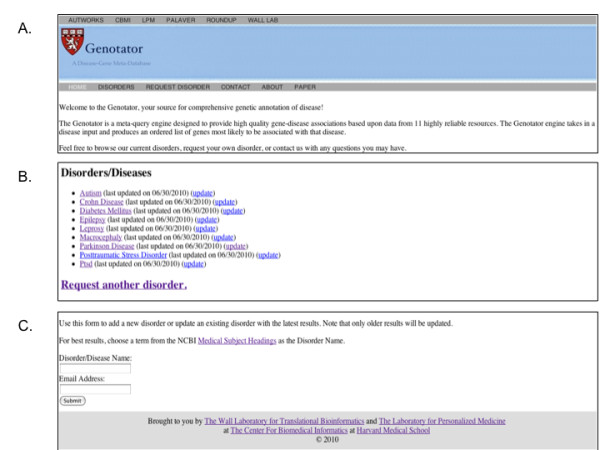
**Screen shot of the main components of Genotator**. Genotator is freely accessible at http://genotator.hms.harvard.edu. (A) The site consists of a splash page, (B) a disorders/diseases page that stores the complete set of results for all queries in alphabetical order, (C) and a query page that accepts a single disease or comma separated disease list and email address for alerting users when the query completes.

We designed a web resource for open access to the Genotator pipeline, available at http://genotator.hms.harvard.edu. The site has two primary components, one for initiating a query through a request form, and another for storing results under a disorder name (Figure 2). Results are stored persistently, together with the creation date. A user may request to update the results for a specific disorder if the results are likely to be outdated based on the last creation date. The request form requires a disorder name, following the standard vocabulary of the Unified Medical Language System, and an active email address. An automatic email is sent to the user when the pipeline completes. The results are then displayed in the "Disorders" tab and saved until updated. Each result set can be displayed in the browser or downloaded in full as a .txt file. The output contains a presence/absence matrix of the databases in which the gene appears; this gives the user the ability to select among the 11 databases and also to rank/reorder the results using criteria other than the Genotator score.

## Results

**Figure 3:**
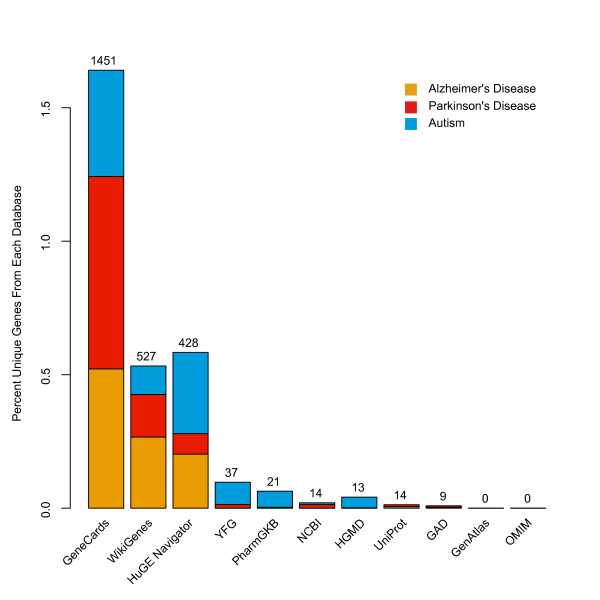
**Percentage of unique contribution made by each of the 11 Genotator resources**. Although a sizeable percentage of results came from GeneCards, WikiGenes, and HugeNavigator (with each contributing well over 200 unique genes totaled across autism, PD, and AD), all but GeneAtlas and OMIM provided at least 9 unique genes. The total number of genes found in each database for the three diseases are listed above the graph. The percent of unique genes was normalized for each disease.

Genotator yielded 663 genes for ASD, 1273 genes for PD, and 2682 genes for AD (full sets of results provided as Additional files 1, 2, and 3). Out of 4618 genes found from all three queries, 77% were reported in GeneCards and 43% in HuGE Navigator, well above the total numbers of genes added by other databases that each contributed less than 31% of the data (Table 3). Nevertheless, all but OMIM and GenAtlas provided at least some non-overlapping gene information. In fact, a total of 2514 genes, 54% of the total found by Genotator, were listed as linked to the disease in only one database (Figure 3), suggesting that their inclusion within the Genotator workflow provided new, and potentially valuable information about the genes involved in human disease.

**Table 3:** The percent contribution of disease candidate genes from each of the 11 databases totaled over autism, Parkinson's Disease, and Alzheimer Disease.

Database	% Contribution
GeneCards	76.85
HuGE Navigator	43.09
WikiGenes	30.51
Genetic Association Database	11.02
UniProt	4.87
Your Favorite Gene	3.23
Human Gene Mutation Database	3.20
PharmGKB	2.88
Online Mendelian Inheritance in Man	2.27
Entrez Gene	1.52
GenAtlas	1.23

### Autism

When using "Autism" as the query term in the Genotator disease search, 663 genes were returned. Six of the top 10 genes returned were listed among the most promising autism candidates in a recent review (Table 4) [11], demonstrating that the top ranked genes by Genotator match recent advances in our understanding of autism genetics. Using our method to rank the 197 SFARI genes, we compared the ordered lists from SFARI and Genotator. Genotator found a total of 77% (152 out of the 197 SFARI 10 genes). All of the top 10 SFARI genes were included in the Genotator set, as well as 19 out of the top 20, 29 out of the top 30, 35 out of the top 40, and 41 out of the top 50.

**Table 4:** Top scoring autism genes ranked on Genotator score.

Gene	Score	Literature Support	Reference	AG Classification
SLC6A4	136.7	Linkage and association analysis at the serotonin transporter (SLC6A4) locus in a rigid-compulsive subset of autism.	[32]	Probable
NRXN1	27.2	Intragenic rearrangements in NRXN1 in three families with autism spectrum disorder, developmental delay, and speech delay.	[33]	Promising
FMR1	22.6	Association and transmission analysis of the FMR1 IVS10 + 14C-T variant in autism	[34]	Probable
PTEN	19.6	Subset of individuals with autism spectrum disorders and extreme macrocephaly associated with germline PTEN tumour suppressor gene mutations.	[35]	Probable
FRAXA*	17.9	Point mutation analysis of the FMR-1 gene in autism.	[36]	
FRAXE*	17.9	Cognitive, behavioral, and neuroanatomical assessment of two unrelated male children expressing FRAXE.	[37]	
FRAXF*	17.3	Mental impairment and attention deficit hyperactivity disorder in a family with FRAXF.	[38]	
CNTNAP2	15.9	Linkage, association, and gene-expression analyses identify CNTNAP2 as an autism-susceptibility gene.	[39,35]	Probable
UBE3A	14.5	Linkage disequilibrium at the Angelman syndrome gene UBE3A in autism families.	[40]	Probable
CDH10	12	Common genetic variants on 5p14.1 associate with autism spectrum disorders.	[41]	

The table lists the title and reference of the research study supporting the gene-disease association together with the official gene symbol. We used the review article by Abrahams and Geschwind (2008) as a source for the most promising autism gene candidates (AG classification). Starred genes are those that did not appear in SFARI Gene. The complete results are available online as Additional file 1.

### Parkinson's Disease

As in Yu et al. 2008, we used the well-known PDGene database as a source for validation of the accuracy and coverage of Genotator. The PDGene database provided a relative ranking (based on HuGENet/Venice grades [10]) that enabled us to determine the extent of overlap with Genotator, both in terms of coverage and strength of association to the disease. Twenty-one of the 32 PDGene "Top Results" genes were in the top 10th percentile of the results returned by Genotator, and in total 58% (312/540 PDGene genes) were found to be in common (Table 5). In addition, 6 of the top 10 Genotator genes were listed among the most promising PD gene candidates in a recent review (Table 5) [12], indicating that Genotator appropriately prioritized leading research in the field.

**Table 5:** Top 10 Parkinson's Disease Genes ranked on Genotator score.

Gene	Score	Literature Support	Reference	WRW Mention
LRRK2	105.1	Frequency of LRRK2 mutations in early-and late-onset Parkinson disease.	[42]	Y
MAPT	60.8	Different MAPT haplotypes are associated with Parkinson's disease and progressive supranuclear palsy.	[43]	Y
SNCA	59.8	Genome-wide association study confirms SNPs in SNCA and the MAPT region as common risk factors for Parkinson disease.	[44]	Y
PARK2	59.6	Case-control study of the parkin gene in early-onset Parkinson disease.	[45]	Y
APOE	34.7	Phenotypic associations of tau and ApoE in Parkinson's disease.	[46]	
GBA	21.4	Genotype-phenotype correlations between GBA mutations and Parkinson disease risk and onset.	[47]	Y
BDNF	19.5	BDNF Val66Met polymorphism is associated with cognitive impairment in Italian patients with Parkinson's disease.	[48]	
DRD2	18.7	Association of DRD3 and GRIN2B with impulse control and related behaviors in Parkinson's disease.	[49]	
MAOB	17.1	Association of variations in monoamine oxidases A and B with Parkinson's disease subgroups.	[50]	
PINK1	16.8	Parkin and PINK1 mutations in early-onset Parkinson's disease: comprehensive screening in publicly available cases and control.	[51]	Y

The table lists the title and reference of the research study supporting the gene-disease association together with the official gene symbol. We used the recent review article by Wider, Ross, and Wszolek (2010) as a source for the most promising Parkinson's disease gene candidates (WRW Mention). A complete set of results is available online as Additional file 2.

### Alzheimer Disease

As a final use case, we focused on AD and the well-known AlzGene database. Similar to the PDGene database, AlzGene provided a relative ranking (based on HuGENet/Venice grades[10]) that enabled us to determine the extent of overlap with Genotator in terms of coverage and strength of association to the disease. Of the three disease use cases, Genotator appeared to perform best with Alzheimer Disease. Thirty-four of the 40 AlzGene "Top Results" genes were in the top 10th percentile of the results returned by Genotator, and in total 74% (486/660) of the candidate genes reported in AlzGene were found by Genotator (Table 6).

**Table 6:** Top 10 Alzheimer Disease Genes ranked on Genotator score.

Gene	Score	Literature Support	Reference
APOE	513	Effect of APOE genotype on amyloid plaque load and gray matter volume in Alzheimer disease.	[52]
PSEN1	45.4	Early Onset Alzheimer's Disease with Spastic Paraparesis, Dysarthria and Seizures and N135 S Mutation in PSEN.	[53]
ACE	28.4	Amyloid-beta-Related Genes SORL1 and ACE are Genetically Associated With Risk for Late-onset Alzheimer Disease in the Chinese Population.	[54]
PRNP	23.1	Earlier onset of Alzheimer's disease: risk polymorphisms within PRNP, PRND, CYP46, and APOE genes.	[55]
BCHE	22.7	Dipeptidyl carboxypeptidase 1 (DCP1) and butyrylcholinesterase (BCHE) gene interactions with the apolipoprotein E epsilon4 allele as risk factors in Alzheimer's disease and in Parkinson's disease with coexisting Alzheimer pathology.	[56]
APOC1	20.8	APOE and APOC1 promoter polymorphisms and the risk of Alzheimer disease in African American and Caribbean Hispanic individuals	[57]
IL1B	19.9	Assessment of Alzheimer's disease case-control associations using family-based methods.	[58]
IL1A	19.5	Pharmacogenomics in Alzheimer's disease.	[59]
MTHFR	19.3	Association of MTHFR gene polymorphism C677T with susceptibility to late-onset Alzheimer's disease.	[60]
BDNF	17.5	Association between BDNF Val66Met polymorphism and Alzheimer disease, dementia with Lewy bodies, and Pick disease.	[61]

The table lists the title and reference of the research study supporting the gene-disease association together with the official gene symbol. A complete set of results is available online as Additional file 3.

## Discussion

As we advance into the era of personalized medicine, our ability to annotate the human genome with clinically actionable information is paramount. An important step in that annotation process is accurate characterization of the genetic etiology of any human disease. Numerous informatics approaches have been and are being developed to assist in this process, including methods for filtering biomedical knowledge for gene-disease association (e.g. [13], [14]), as well as full scale natural language processing approaches [15,16], although the corpora necessary for both high precision and recall remain incomplete [16]. As these strategies have matured, an abundance of databases have emerged to provide summaries of recent and historical advances in human disease research. However, because these databases differ in their coverage of genes and annotation content, it is challenging for a researcher to develop a complete picture for a single disease or set of diseases of interest. In order to facilitate multi-database searching and to provide a more complete picture of advances in genetic research of human diseases, we developed a software tool called "Genotator". Genotator generates a comprehensive set of results for any disease by integrating gene and annotation data from 11 externally accessible and best-of-breed genetic resources. The results from Genotator are ranked using a scoring system that integrates bibliomic and genomic data and provides a preliminary likelihood of strength of association for use in future thesis testing.

To test the content of Genotator and assess the efficacy of its scoring system, we applied Genotator to three separate diseases: Autism Spectrum Disorder (ASD), Parkinson's Disease (PD) and Alzheimer Disease (AD), and compared our results to the three web resources devoted to manual curation of these diseases, SFARI gene, PDgene, and AlzGene, respectively. Genotator's results were in high agreement, with over 75% in common with the gene lists provided by SFARI gene for autism, nearly 60% in common with the PDGene for PD and almost 75% in common with AlzGene for AD. Furthermore, the rank order provided by Genotator matched the prioritizations by these resources, especially among the most highly 12 ranked cohort of genes, supporting the notion that Genotator provides similar coverage and quality to that available from manually curated, well-funded resources that are under active development.

In addition, we were able to demonstrate the value-add provided through integration of the 11 different resources used by Genotator. Nearly every database reported genes not reported by any of the other databases, replete with sufficient justification for the gene-disease link (Figure 3). Thus, Genotator can achieve algorithmically what would otherwise require extensive manual labor. Genotator also provides an enriched output with features often lacking from other disease annotation including synonym disambiguation, standard HGNC nomenclature, and the ability to download the entire dataset including annotations, pubmed ids, and scores.

As the boundaries between diseases become more obscured, and as our definitions evolve in the wake of new genetic information, resources that provide high coverage of human disease are becoming increasingly more important. While Genotator will not obviate the need for manually curated disease-specific databases going forward, it will enable researchers to keep pace with the research being done on their disease of interest, including those for which devoted websites do not currently exist.

## Conclusions

Genotator is a comprehensive biomedical informatics tool that integrates over a host of mainstream databases to provide automatic and up-to-date information on any human disease. Based on our analysis using three well-studied disorders, we confirmed that the results generated by Genotator match the quality and coverage of manually curated and disease-specific databases. This outcome, coupled with the highly flexible and detailed output, make Genotator a novel and valuable contribution to the field.

## Availability and Requirements

**Software Name**: Genotator

**Project home page**: http://genotator.hms.harvard.edu

**Programming Languages**: Java, Python; source code available by request

## Competing interests

The authors declare that they have no competing interests.

## Authors' contributions

DPW conceived the study, participated in algorithm design, analysis, and code development, and wrote the manuscript. RP designed and validated the algorithms, participated in code development, and wrote the manuscript. MT implemented the pipeline with assistance from DPW and JYJ. VF assisted in algorithm development and manuscript preparation. TFD developed the website and edited the manuscript. PJT conceived the study, assisted in algorithm design and analysis, and participated in the writing. All authors read and approved the final document.

## Pre-publication history

The pre-publication history for this paper can be accessed here:

http://www.biomedcentral.com/1755-8794/3/50/prepub

## Supplementary Material

Additional file 1 **Complete Genotator results for autism**. Contains the complete set of genes identified by Genotator as linked to autism together with the full set of attributes gathered by the Genotator resource (Figure 1). Click here for file

Additional file 2 **Complete Genotator results for Parkinson's Disease**. Contains the complete set of genes identified by Genotator as linked to Parkinson Disease together with the full set of attributes gathered by the Genotator resource (Figure 1). Click here for file

Additional file 3 **Complete Genotator results for Alzheimer Disease**. Contains the complete set of genes identified by Genotator as linked to Alzheimer Disease together with the full set of attributes gathered by the Genotator resource (Figure 1). Click here for file
